# Supplementary material for: Adapting the Community-based Health Planning and Services (CHPS) to engage poor urban communities in Ghana: protocol for a participatory action research study
Source: BMJ Open. 2021 Jul 27;11(7):e049564. doi: 10.1136/bmjopen-2021-049564 (PMC8317127; doi:10.1136/bmjopen-2021-049564)
Supplement: Author's manuscript [file bmjopen-2021-049564.draft_revisions.pdf]

BMJ Open is committed to open peer review. As part of this commitment we make the peer review history of every article we publish publicly available.

When an article is published we post the peer reviewers' comments and the authors' responses online. We also post the versions of the paper that were used during peer review. These are the versions that the peer review comments apply to.

The versions of the paper that follow are the versions that were submitted during the peer review process. They are not the versions of record or the final published versions. They should not be cited or distributed as the published version of this manuscript.

BMJ Open is an open access journal and the full, final, typeset and author-corrected version of record of the manuscript is available on our site with no access controls, subscription charges or pay-per-view fees (http://bmjopen.bmj.com).

If you have any questions on BMJ Open's open peer review process please email info.bmjopen@bmj.com

BMJ Open

Adapting the community-based health planning and services (CHPS) to engage urban poor communities in Ghana: protocol for a participatory action research study.

Journal:	*BMJ Open*
Manuscript ID	bmjopen-2021-049564
Article Type:	Protocol
Date Submitted by the Author:	26-Jan-2021
Complete List of Authors:	Abboah-Offei, Mary; University of York<br>Gyasi, Akosua; Ghana College of Physicians and Surgeons<br>Ayim, Andrews; Ghana College of Physicians and Surgeons<br>Ansah-Ofei, Adelaide; University of Ghana, School of Nursing and Midwifery<br>Dovlo, Delanyo; Ghana College of Physicians and Surgeons<br>Awoonor-Williams, John ; Ghana Health Service<br>Agongo, Erasmus; Ghana College of Physicians and Surgeons<br>Agyepong, Irene; Ghana Health Service<br>Elsey, Helen ; University of York,
Keywords:	PUBLIC HEALTH, HEALTH SERVICES ADMINISTRATION & MANAGEMENT, PRIMARY CARE, SOCIAL MEDICINE

SCHOLARONE™
Manuscripts

I, the Submitting Author has the right to grant and does grant on behalf of all authors of the Work (as defined in the below author licence), an exclusive licence and/or a non-exclusive licence for contributions from authors who are: i) UK Crown employees; ii) where BMJ has agreed a CC-BY licence shall apply, and/or iii) in accordance with the terms applicable for US Federal Government officers or employees acting as part of their official duties; on a worldwide, perpetual, irrevocable, royalty-free basis to BMJ Publishing Group Ltd ("BMJ") its licensees and where the relevant Journal is co-owned by BMJ to the co-owners of the Journal, to publish the Work in this journal and any other BMJ products and to exploit all rights, as set out in our licence.

The Submitting Author accepts and understands that any supply made under these terms is made by BMJ to the Submitting Author unless you are acting as an employee on behalf of your employer or a postgraduate student of an affiliated institution which is paying any applicable article publishing charge ("APC") for Open Access articles. Where the Submitting Author wishes to make the Work available on an Open Access basis (and intends to pay the relevant APC), the terms of reuse of such Open Access shall be governed by a Creative Commons licence – details of these licences and which Creative Commons licence will apply to this Work are set out in our licence referred to above.

Other than as permitted in any relevant BMJ Author's Self Archiving Policies, I confirm this Work has not been accepted for publication elsewhere, is not being considered for publication elsewhere and does not duplicate material already published. I confirm all authors consent to publication of this Work and authorise the granting of this licence.

For peer review only

Adapting the community-based health planning and services (CHPS) to engage urban poor communities in Ghana: protocol for a participatory action research study.

Abboah-Offei, M[1]., Gyasi, A.[2], Ayim, A.[3], Ansah-Ofei, A. M.[4], Dovlo, D.[5], Awoonor-Williams J. K.[6], Agongo E. E. A[7], Agyepong, I. A.[8] and Elsey, H.[9]

Author details:

1. Dr Abboah-Offei, Mary; Department of Health Sciences, University of York Heslington, York Y10 5DD email: mary.abboah-offei@york.ac.uk
2. Dr Gyasi, Akosua; Ghana College of Physicians and Surgeons, Public Health Faculty P.O. Box MB 429, Ministries, Accra, Ghana email: akosua.gyasi@yahoo.com
3. Dr Ayim, Andrews; Address: Ghana College of Physicians and Surgeons, Public Health Faculty P.O. Box MB 429, Ministries, Accra, Ghana Mobile: +233244230466 email: andyayim@yahoo.co.uk
4. Dr Ansah-Ofei, Adelaide Maria; School of Nursing and Midwifery, University of Ghana, Legon Tel. 0244653065/0204653065 email: adelaideofei@gmail.com
5. Dr Dovlo, Delanyo; Ghana College of Physicians and Surgeons, Public Health Faculty P.O. Box MB 429, Ministries, Accra, Ghana email: dovlod@gmail.com
6. Dr Awoonor-William, John Koku; Director, Policy Planning Monitoring and Evaluation, Ghana Health Service Private Mail Bag, Ministries, Accra, Ghana Tel: +233-208161394 +233 244564120 email: kawoonor@gmail.com
7. Dr Agongo, Erasmus Emmanuel Akurugu; Ghana College of Physicians and Surgeons, Public Health Faculty P.O. Box MB 429, Ministries, Accra, Ghana email: erasmusagongo@yahoo.co.uk
8. Professor Agyepong, Irene Akua; Ghana Health Service Research and Development Division Dodowa Health Research Center, P.O. Box DD 1, Dodowa. Tel. 233-244 862 665 /233-208 133 850; email: irene.agyepong@gmail.com.
9. Dr Elsey, Helen; Department of Health Sciences, University of York Heslington, York Y10 5DD Tel: 0790 4681241 email: helen.elsey@york.ac.uk

For peer review only

Corresponding author:

Mary Abboah-Offei

Department of Health Sciences
University of York
Heslington, York YO10 5DD

E-mail: mary.abboah-offei@york.ac.uk
Tel. 07500045742

For peer review only

For peer review only - http://bmjopen.bmj.com/site/about/guidelines.xhtml

Abstract

Introduction With rapid urbanisation in low- and middle-income countries, health systems are struggling to meet the needs of their growing populations. Community-based Health Planning and Services (CHPS) in Ghana have been effective in improving maternal and child health in rural areas however, implementation in urban areas has proved challenging.

Aims To engage community health officers, volunteers and communities to understand their social structures, needs, health seeking and health insurance behaviour, and to identify and specify system adaptations required to effectively implement CHPS sustainably and at-scale to reach the urban poor communities in Ghana.

Methods and analysis A Participatory Action Research (PAR) will be used to develop an urban CHPS model with stakeholders in three selected CHPS zones (a. Old Fadama (Yam and Onion Market community), b. Adedenkpo and c. Adotrom 2) representing three categories of poor urban neighbourhoods in Accra, Ghana. Two phases will be implemented: Phase one ('Reconnaissance phase) will engage and establish PAR research groups in the selected zones, conduct focus groups and individual interviews with urban residents, households vulnerable to ill-health and CHPS staff and key stakeholders. A desk review of preceding efforts to implement CHPS will be conducted to understand what worked (or not), how and why. Findings from Phase one will be used to inform and co-create an urban CHPS model in Phase two, where PAR groups will be involved in multiple recurrent stages (cycles) of community-based planning, observation, action and reflection to develop and refine the urban CHPS model. Data will be managed using NVivo software and coded using the domains of community engagement as a framework to understand community assets and potential for engagement.

Ethics and dissemination Ethical approval has been granted in the United Kingdom and Ghana. The results of this study will guide the scale-up of CHPS across urban areas in Ghana, which will be disseminated through journal publications, community and government stakeholder workshops, policy briefs and social media content. This study is also funded by the Medical Research Council, United Kingdom.

Keywords: Ghana, CHPS, Urban communities, Community engagement, Participatory Action Research

Word count: 3,604

For peer review only

Article summary:

Strengths and limitations of this study

- Close engagement with Ghana Health Service and communities throughout the study will enable the development of an urban CHPS model that can be delivered sustainably within the current health system.
- Using PAR will enable increased engagement and the collaboration with research participants and stakeholders.
- The mixed methods used within the PAR approach in three different urban poor neighbourhoods will provide in-depth understanding of the health needs of vulnerable urban residents.
- PAR is time intensive and will require prolonged engagement with the research setting and stakeholders.
- Given the level of engagement involving the study, the COVID-19 pandemic presents a major risk to our ability to implement the study as planned.

For peer review only

Introduction

Sub Saharan Africa is urbanising fast; with over half of the continent's population predicted to live in urban areas by 2035[1]. Governments, at national and city level, face multiple challenges in addressing the growing health needs of their expanding populations[2]. Despite outdated notions of an 'urban advantage', proximity to healthcare does not equate to access to free, high quality healthcare services or health promoting interventions for the urban poor[3]. This often result in worse outcomes than their rural counterparts and better-off urban residents[4]. Ghana is one of the most urbanised countries in sub-Saharan Africa with 56.7% of the population estimated to be living in urban areas in 2019[5]. These rapidly expanding cities and towns are characterized by slum and peri-urban communities with poor infrastructure, over-crowded and unsanitary conditions, which increases the risk for both communicable and non-communicable diseases, resulting in inequalities, poverty and marginalization[2,6]. With public services struggling to reach urban poor communities, people turn to a range of predominantly private clinics and pharmacies, however, with limited uptake of health insurance by the poorest [6] their access to quality and affordable health-care services and health promoting activities are severely limited. This has impacted on infant and child mortality which are five times higher in poor urban communities compared to the general urban population [6]. Ensuring appropriate, quality service delivery to households will improve timely, suitable care and health promotion activities and reduce vulnerability to expensive and inappropriate care delivery through a plethora of unregulated providers.

Ghana's three-tier district health system has at its foundation the Community-based Health Planning and Services (CHPS) programme, which has been successfully delivering universal access to health promotion, prevention and basic curative care in rural districts using community-based nurses known as Community Health Officers (CHOs) and volunteers [7-16]. This has led to a reduction in childhood mortality by a third [17] and decline in total fertility by one birth [18]. However, despite government policy to scale-up CHPS nationally, these benefits do not currently extend to the urban population [19,20]. CHPS implementation in urban areas of Ghana has been limited to a few pilot districts [7] and there are calls for more research to inform an urban CHPS model [8]. Evidence from CHPS piloted in some urban areas have revealed a need for greater range of services including improved approaches to the delivery of the Integrated Management of Childhood Illnesses (IMCI); sustained and expanded engagement of communities and volunteering programmes; improved motivation and skills training for staff and opportunities for career progression [20]. Limited registration with Ghana's National Health Insurance Scheme (NHIS) further undermines access to health services in urban areas [21]. NHIS was created in 2003 with the ideal of being 'pro-poor', however, recent studies have shown only 17% of the poor are insured compared to 44% among rich households [22]. Although those classified as extremely poor automatically qualify for a free NHIS card [23], the extent to which this is taken-up in poor urban areas is unknown. Developing appropriate and sustainable system-wide solutions in the urban context is vital if urban CHPS is to move beyond a few pilot areas.

For peer review only - http://bmjopen.bmj.com/site/about/guidelines.xhtml

Community engagement is a central pillar of CHPS with six milestones of implementation[8], however in many poor urban areas social structures and cohesion look very different from those found in rural areas. Urban poor neighbourhoods are frequently characterised transient migrant populations and both men and women working long hours with reduced support from extended family and multiple stresses of urban living [24]. Studies have identified particular challenges in engaging urban communities in health programmes and activities [25-27]. Understanding how best to engage urban poor households, particularly the most vulnerable women and children is vital for effective adaptation and scale up of CHPS within urban areas. Methods for community engagement in decisions that affect their lives have been criticized for the lack of participatory approaches and inability to move beyond tokenistic mode of participation [28-30]. One approach to addressing these criticisms is Participatory Action Research (PAR) [31,32], which will help to identify and specify system adaptations required to effectively implement CHPS sustainably to reach the urban poor. The PAR approach will enable meaningful engagement with stakeholders, communities and vulnerable households in identifying social structures, health needs and other areas of concern through collaboration and capacity building to address these issues holistically [33-35].

In order to enhance a better understanding of engagement within health systems, this study has adopted the conceptual framework derived from a systematic review of public health interventions (see Figure 1)[36]. This framework provides clarity on aspects of engagement from a community and a health system perspective, and has the advantage of being an empirically driven model utilising findings from both qualitative and quantitative studies[36]. Although studies included in this review were predominantly from high-income countries, the insights show similarities to reviews of engagement in low and middle-income countries (LMICs) [25-27]. This study aims to engage CHOs, volunteers and communities to understand their social structures, needs, health seeking and health insurance behaviour, and to identify and specify system adaptations required to effectively implement CHPS sustainably and at-scale to reach the urban poor in Ghana.

Insert Figure 1: A conceptual framework for community engagement in interventions Brunton et al. [36].

Study objectives

1. To explore social structures of poor urban communities, and vulnerable individuals and households, including the uninsured, and identify their current health seeking behaviour, using this information to adapt the CHPS model.

For peer review only - http://bmjopen.bmj.com/site/about/guidelines.xhtml

2. To review and critically analyse preceding efforts to implement rural and urban CHPS to understand what worked (or not), how and why.
3. To design with urban communities, CHPS community teams and health system managers, an adaptation of the current CHPS programme and system to ensure urban relevance.
4. To evaluate early implementation processes, costs, and acceptability from the perspective of urban populations who are marginalized and vulnerable such as women and children and frontline health workers, volunteers and health systems managers and identify the pathway for scale up.
5. To make recommendations on contextually appropriate modifications to the CHPS and NHIS policy, programme and implementation arrangements for urban localities as well as approaches to scale up

Methods

Study design

The study uses both quantitative and qualitative methods within the overall approach of participatory action research (PAR). PAR is a rigorous and systematic approach to enquiry, which allows researchers and stakeholders to explore and discover effective solutions to life problems [37]. PAR gives stakeholders the opportunity to be involved with multiple recurrent stages (cycles) of community-based planning, action, observation and reflection[38] with each cycle following on from and influencing subsequent cycles [37,39]. The use of PAR in the community is beneficial in increasing engagement and the collaborative nature of the study [40,41]. We chose PAR as an appropriate methodology for this study as it will enable us to try out different approaches to engagement in a range of urban poor settings and reflect on the experience in collaboration with CHOs, volunteers and community members.

In addition, given that community engagement is a key component of CHPS, the alignment of PAR to community engagement will help to strengthen CHPS staff skills in participation approaches, whilst simultaneously strengthening engagement bonds with poor urban communities. This level of engagement we hope will increase community ownership of the urban CHPS model, supporting scale-up and sustainability. For example, we will clarify, from the perspective of communities and CHPS, which population groups and communities should be targeted and understand the motivations, both from communities and CHPS, for their inclusion. This will include understanding the motivations for registering (or not) for NHIS and facilitating those excluded from NHIS to identify ways to encourage registration among these groups. We will also explore the extent of participation, the mediators and context for engagement. Our PAR cycles will allow us to consciously address process issues and document the successes for different approaches to process. We will explore the impact of community engagement on participants, the wider community, within the CHPS teams

For peer review only - http://bmjopen.bmj.com/site/about/guidelines.xhtml

and the health system (both potential benefits and harms). This study will be implemented in two phases: Phase one will address study objectives 1 to 2, which will constitute 'Reconnaissance phase' in PAR; and Phase 2 will address study objectives 3 to 5 and will comprise a number of PAR cycles (see Figure 2).

Setting

We purposively selected three poor urban CHPS zones: (a) Old Fadama (Yam and Onion Market community), (b) Adedenkpo and (c) Adotrom 2) that do not currently have functional CHPS programme (i.e. a designated CHPS zone that is lacking in facilities, staff or targeted outreach services), and with differing characteristics, to allow for transferability across urban areas of differing social characteristics. A CHPS zone includes about 5,000 to 10,000 population assigned to a Community Health Team led by a CHO. CHPS zones are linked to primary health clinics/maternity homes as the next level of care, and then to District/Municipal Hospital and Health Management Team. This three-tier system allows for referral as well as supervision and monitoring from the community level. The three selected zones will include: (a) an informal settlement of predominantly first-generation migrants; (b) a mixed poor/better-off neighbourhood; and (c) a long-established neighbourhood of several generations. We will also select different levels of CHPS functionality (e.g. with facilities and staff but no volunteers or outreach, or with no facilities or staff in the demarcated zone but with support from neighbouring zones).

STUDY PHASE 1

Reconnaissance phase

The 'Reconnaissance Phase' [42] is described in the action research literature as an observational phase to gain insight into the problem and develop a theorised-account to inform action[42]. The information generated in the reconnaissance phase will inform and enable the establishment of groups of co-researchers in each of the three CHPS zones. The findings of the reconnaissance phase will also inform that CHPS models to be tried in the first PAR cycle in each CHPS zone. PAR groups will be made up of key stakeholders, or co-researchers, affected by the health and access problems experienced in their neighbourhoods. The co-research groups will include community members, CHPS staff, volunteers and mangers. The reconnaissance phase enables theory building within action-research[42], emerging theories will be structured according to the theoretical framework in Figure 1 [36]. We will also be mindful of emerging aspects not reflected in the framework; this is particularly important given the limited LMIC-based studies that have informed the framework. During PAR cycles in study phase 2, the three groups of co-researchers will work through the cycles of 'plan, act, observe, reflect' [31] (see Figure 2) in their respective CHPS zones, developing the model.

Phase 1 (Reconnaissance Phase)

For peer review only - http://bmjopen.bmj.com/site/about/guidelines.xhtml

This initial phase will focus on consultations with Greater Accra Regional Health Directorate and Regional/ District CHPS coordinators to select 3 CHPS zones for the study; engagement with the local team (CHO, CHN, CHN & managers) in each zone; checking and building capacity of community members, key stakeholders & CHPS staff to commit to the study in terms of knowledge on PAR, availability to attend training on PAR or train others to become PAR co-researchers. This phase will address research objectives 1 and 2, enabling us to find a shared concern among key stakeholders and community members in their respective CHPS zones to begin to identify solutions from the perspective of those most affected [39], particularly community members vulnerable to ill-health and poor access to health services and CHPS staff and volunteers.

To answer objective 2 and enable understanding of the key features, mechanisms and outcomes of the CHPS programme, we will conduct a mixed methods review using a results-based convergent design[43], where the quantitative and qualitative findings are synthesised separately and then brought together in a final synthesis. This will allow us to synthesise quantitative results from included studies on the outcomes of CHPS and findings from qualitative, mixed-methods or quantitative studies on the mechanisms (e.g. health system, participant or contextual factors) that may influence effectiveness. We will include any study evaluating the CHPS programme in urban or rural Ghana and will particularly look to identify facilitators and barriers to success in urban contexts.

We will also work closely with Ghana Health Service and the CHPS programme, taking an embedded research approach[44] to ensure that issues of sustainability and scale-up are central to the design. We will also establish the groups of co-researchers in each CHPS zones, share the findings form the reconnaissance work, and facilitate discussions on adaptations of the CHPS model to respond to community needs.

Insert Figure 2: Proposed PAR activities and cycles for this study

Recruitment and data collection in Phase 1

A purposive sample of key health providers and community stakeholders will be recruited from the three selected zones for an initial focus group discussion. Two focus groups (FGs) each will be conducted in each zone using techniques such as 'social-mapping' to facilitate discussion on social structures and health seeking behaviour, and 'chapati diagrams' [45] to identify health providers and their relative importance (each FG will comprise of 8 to 10 participants, and a total of n=6 FGs will be held). Local gatekeepers from the FGs will help researchers to identify particularly vulnerable household (e.g. with under-5 children, female-headed, elderly or chronically ill or disabled) for individual interviews to explore the challenges they face in registering for insurance, keeping healthy and seeking care. Eight individual interviews will be conducted in each zone making a total of n=24 interviews informing 'definitions' and 'motivations' within the framework [36]. The sequential follow-up

For peer review only - http://bmjopen.bmj.com/site/about/guidelines.xhtml

of individual interviews is aimed at gaining a deeper understanding of the internal and external issues and concerns surrounding the implementation and use of CHPS services.

Data will be collected on baseline costs and utilisation, disaggregated by gender and age, of any elements of from existing Urban CHPS pilot. The findings of the mixed-methods systematic review of the CHPS programme in rural and urban areas will be used to inform key informant individual interviews with CHPS staff and volunteers (n=10) and community members (n=10) in the three selected zones. These interviews will explore all six aspects of the framework [36] in Figure 1.

Data analysis for Phase 1

All interviews and focus groups will be audio recorded and transcribe as soon after data collection as possible. Transcripts will be translated into English. Data collected will be reviewed and coded using the Brenton et al domains of community engagement as a framework to understand community assets and potential for engagement. The data collected in phase one will also enable identification of the needs, gaps, weaknesses and opportunities within the three selected zones relating to CHPS, staff and volunteers, community members and other relevant stakeholders. We will follow the seven stages within Framework Approach as described by Gale et al[46]. Data will be managed using NVivo software. This analysis will inform the initial CHPS models considered by the co-researchers in each zone.

Throughout the study, the research team will keep a comprehensive reflective research journal, which will be used to catalogue the progress, obstacles and successes of the PAR process. This journal will be kept to acknowledge the research team's experience of PAR in the urban context, analysis and interpretation [47,48]. The journal will also act as a component of the audit trail for the study [49]. Reflective journals also increases external validity by making subjective processes transparent [48]. During this phase the CHPS staff, volunteers and researchers will receive training and support on participatory methods and the principles underpinning PAR : participation: "Action research is only possible with, for and by persons and communities, ideally involving all stakeholders both in the questioning and sense making that informs the research, and in the action which is its focus"[50]; and produce practical knowledge, and to do that draws on representational, relational and reflective knowledge and is for a worthwhile purpose[50].

STUDY PHASE 2: PAR Cycles

The PAR co-researchers (PAR groups) will work through PAR cycles to co-create the urban CHPS model with stakeholders, implement and evaluate the processes, cost and acceptability of this model from the perspectives of the urban poor population. To initiate this process, initial stakeholder meetings will be held with the PAR groups in each of the three zones to co-create an urban CHPS

For peer review only - http://bmjopen.bmj.com/site/about/guidelines.xhtml

model drawing on the findings from Phase 1. The emerging understanding and findings from Phase 1 in relation to the conceptual framework[36] adopted (see Figure 1) will be discussed with the PAR groups to trigger and inform the design of the prototype urban CHPS model. Different design options will be costed to enable consideration of sustainability and scale-up at this early stage.

Following from the workshop with the PAR groups from the three zones, the co-researchers in each zone will begin the PAR cycles. The PAR groups will be facilitated by public health professionals in training (residents) from the Ghana College of Physicians and Surgeons (GCPS), who will also document the decisions taken by the PAR groups and support them to identify the most effective methods for assessing whether the adaptations to the CHPS model work in practice in increasing access, feasibility and appropriateness of CHPS in their community. This observation stage will include use of routine health information data to understand patterns of utilisation of CHPS services disaggregated by gender, age and diagnosis (variables routinely available in the District Health Information Management Systems (DHIMS)). The PAR groups will monitor utilisation data on an on-going basis identifying those excluded and responding by reshaping aspects of the model in subsequent PAR cycles (see Figure 2).

The co-researchers and public health residents will also use qualitative methods including observation, interviews and focus groups as appropriate to understand issues of acceptability, feasibility and access in more depth and from the perspective of those most likely to struggle in accessing health care and improving health. Actual costs of delivering the model from the health service perspective in the different urban zones will be collected. This will include costs of staff and volunteer training, time and salary or expenses, transportation, materials and equipment, supervision. The utilisation data will be used to estimate the cost per patient in each of the three zones.

Emerging findings from the co-researchers in the three zones will be discussed with GHS and CHPS programme managers over the 12 months period of implementation of the PAR cycles. At the end of the 12 months, a workshop will be held with CHPS managers, provincial and national health system stakeholders to assess the findings form the 3 zones and plan the next steps in implementation and scale-up, if appropriate.

We will develop guidelines, training and standard operating procedures, recording and reporting formats for the urban CHPS programme based on study findings.

Patient and public involvement

Community members from the selected CHPS zones will be involved throughout the study. The community leaders and the CHPS managers in these selected communities will assist in negotiating access to the community. This will include local recruitment of PAR co-researchers and hosting of

For peer review only - http://bmjopen.bmj.com/site/about/guidelines.xhtml

series of community engagement meetings with PAR groups where the prototype urban CHPS model design will be discussed
and later results will be disseminated.

Rigour

Appropriate measures will be implemented to increase the rigour of this study. Data will be collected and coded by the research team and discussions held regularly with stakeholders to reduce bias [51]. Sources of potential bias that could influence the processes of data collection and analysis due to existing networks and connections will be acknowledged and recorded. This level of documentation will increase confirmability by providing an audit trail, which will allow observers to confirm the veracity of the study [37,52]. Prolonged engagement with the community and stakeholders will increase credibility and regular member checking of raw data, analyses and reports [37]. Transferability will be provided through detailed descriptions of the contextual data and activities of the study, through immersion, reflective journaling and detailed documentation, this will allow other researchers to analyse the situation and study outcomes based on context [52,53].

The coding and themes will be analysed by at least 2 to 3 members of the research team to enhance credibility[54]. This will involve a reflective practice whereby the team leader will first code the data, and then these codes will be discussed by the research team and further refined to ensure the codes fit with the framework and any emerging themes reflect the dataset. This process will enhance dependability and inter-coder reliability[54]. The research team will be involved in the development of all interview guides and further refinement of the guide will occur as a team.

Ethics approval

This study has been approved by the University of York's Health Sciences Research Governance Committee (HSRGC/2020/409/E) and the Ghana Health Service Ethics Review Committee (GHS-ERC 003/10/20). Given the community participatory nature of this study, there are ethical considerations in relation to protecting the anonymity of participants and confidentiality of data, particularly regarding interviews and focus groups. The connected nature of community members will be acknowledged in consent forms, ground-rules of confidentiality will be agreed in all group discussions, care will be taken in analysis, and presentation of data in ensuring that participant confidentiality is protected. Data that may overtly identify participants will be excluded [55]. Consent will be required from all participants prior to their participation in the study.

Capacity building and dissemination

This study aims to develop a centre of excellence for training and build capacity for Urban CHPS, which will develop and deliver training for CHPS urban teams on clinical and participatory techniques and processes. Under guidance of the local research team in Ghana, public health registrars training under the GCPS will be seconded to the centre evaluating, developing and supporting CHPS

For peer review only - http://bmjopen.bmj.com/site/about/guidelines.xhtml

throughout scale-up. The PAR process will build capacity of CHPS workers in participatory approaches and use of DHIMS data to develop strategies to reach those who may struggle to access CHPS services.

The results of this study will guide the scale-up of CHPS across urban areas in Ghana providing detailed information on all components, costs and impact on utilisation, with outputs such as training materials, operational guidelines and policy revisions. This will lay the foundation for a community-driven model that fits sustainably within the GHS. The centre of excellence will facilitate further evaluation and sharing of good practice. Beyond Ghana, this study, and the planned follow-on evaluation of scale-up will provide much needed evidence and insight into how to engage communities in urban areas so their needs are addressed appropriately by the health system. Policymakers, practitioners and researchers involved in urban health across LMICs are grappling with these issues and searching for solutions to improve the health of the poorest urban residents.

Acknowledgements

We acknowledge all authors for their contribution to this protocol.
We also acknowledge the University of York, Ghana College of Physicians and Surgeons and the Ghana Health Service for supporting this study. Finally, we acknowledge the Medical Research Council for funding this study.

Author contributions

AEEA, EH, AIA, KJA-W developed the concept for the study and successful sought funds from Medical Research Council to conduct the study. MA-O developed the manuscript with inputs from all other authors.

Funding statement

The Medical Research Council grant number MR/T022787/1 support this work.

Competing interest statement

None

For peer review only - http://bmjopen.bmj.com/site/about/guidelines.xhtml

References

1. UN DoE, Social Affairs PD. World Urbanization Prospects: The 2018 Revision (ST/ESA/SER. A/420): United Nations New York; 2019.
2. Elsey H, Agyepong I, Huque R, et al. Rethinking health systems in the context of urbanisation: challenges from four rapidly urbanising low-income and middle-income countries. *BMJ Glob Health.* 2019;4(3):e001501.
3. Shawar YR, Crane LG. Generating global political priority for urban health: the role of the urban health epistemic community. *Health policy and planning.* 2017;32(8):1161-1173.
4. Mberu BU, Haregu TN, Kyobutungi C, Ezeh AC. Health and health-related indicators in slum, rural, and urban communities: a comparative analysis. *Global health action.* 2016;9(1):33163.
5. UNData. Ghana. 2019; https://data.un.org/en/iso/gh.html. Accessed October 14, 2020.
6. Taylor P, Boussen CR, Awunyo-Akaba J, Nelson J. Urban Health Assessment. *Prepared for the USAID Mission to Ghana, Activity Report.* 2002;114.
7. Johnson FA, Frempong-Ainguah F, Matthews Z, et al. Evaluating the impact of the community-based health planning and services initiative on uptake of skilled birth care in Ghana. *PLoS One.* 2015;10(3):e0120556.
8. Nyonator FK, Awoonor-Williams JK, Phillips JF, Jones TC, Miller RA. The Ghana community-based health planning and services initiative for scaling up service delivery innovation. *Health policy and planning.* 2005;20(1):25-34.
9. Awoonor-Williams JK, Sory EK, Nyonator FK, Phillips JF, Wang C, Schmitt ML. Lessons learned from scaling up a community-based health program in the Upper East Region of northern Ghana. *Global Health: Science and Practice.* 2013;1(1):117-133.
10. Awoonor-Williams JK, Bawah AA, Nyonator FK, et al. The Ghana essential health interventions program: a plausibility trial of the impact of health systems strengthening on maternal & child survival. *BMC health services research.* 2013;13(S2):S3.
11. Awoonor-Williams JK, Appiah-Denkyira E. Bridging the intervention-implementation gap in primary health care delivery: the critical role of integrated implementation research. *BMC Health Services Research.* 2017;17(3):91-94.
12. Nyonator FK, Akosa AB, Awoonor-Williams JK, Phillips JF, Jones TC. Scaling up experimental project success with the Community-based Health Planning and Services initiative in Ghana. *Scaling up health service delivery: from pilot innovations to policies and programmes. Geneva: WHO.* 2007:89-112.
13. Awoonor-Williams J, Feinglass E, Tobey R, Vaughan-Smith M, Nyonator F, Jones T. The impact of a replication project on safe motherhood and family planning behavior in Nkwanta District of Ghana. *Studies in Family Planning.* 2004;35:161-177.

For peer review only - http://bmjopen.bmj.com/site/about/guidelines.xhtml

14. Ngom P, Debpuur C, Akweongo P, Adongo P, Binka FN. Gate-keeping and women's health seeking behaviour in Navrongo, northern Ghana. *African journal of reproductive health.* 2003:17-26.
15. Binka FN, Nazzar A, Phillips JF. The Navrongo community health and family planning project. *Studies in family planning.* 1995:121-139.
16. Atinga RA, Agyepong IA, Esena RK. Ghana's community-based primary health care: why women and children are 'disadvantaged'by its implementation. *Social Science & Medicine.* 2018;201:27-34.
17. Pence B, Nyarko P, Binka FN, Phillips JF, Debpuur C. The impact of the Navrongo Community Health and Family Planning Project on child mortality, 1993–2000. Paper presented at: Global Conference of the International Union for the Scientific Study of Population, Salvador, Brazil, August2001.
18. Debpuur C, Phillips JF, Jackson EF, Nazzar A, Ngom P, Binka FN. The impact of the Navrongo Project on contraceptive knowledge and use, reproductive preferences, and fertility. *Studies in family planning.* 2002;33(2):141-164.
19. Adongo PB, Phillips JF, Aikins M, et al. Does the design and implementation of proven innovations for delivering basic primary health care services in rural communities fit the urban setting: the case of Ghana's Community-based Health Planning and Services (CHPS). *Health research policy and systems.* 2014;12(1):16.
20. Nwameme AU, Tabong PT-N, Adongo PB. Implementing community-based health planning and services in impoverished urban communities: health workers' perspective. *BMC health services research.* 2018;18(1):1-11.
21. Kwarteng A, Akazili J, Welaga P, et al. The state of enrollment on the National Health Insurance Scheme in rural Ghana after eight years of implementation. *Int J Equity Health.* Dec 31 2019;19(1):4.
22. Kotoh AM, Aryeetey GC, Van Der Geest S. Factors that influence enrolment and retention in Ghana'National Health Insurance Scheme. *International journal of health policy and management.* 2018;7(5):443.
23. Handa S, Park M, Darko RO, Osei-Akoto I, Davis B, Daidone S. Livelihood empowerment against poverty program impact evaluation. *Chapel Hill: Carolina Population Center, University of North Carolina.* 2013.
24. Lyons M, Snoxell S. Creating urban social capital: some evidence from informal traders in Nairobi. *Urban Studies.* 2005;42(7):1077-1097.
25. Atkinson J-A, Vallely A, Fitzgerald L, Whittaker M, Tanner M. The architecture and effect of participation: a systematic review of community participation for communicable disease control and elimination. Implications for malaria elimination. *Malaria journal.* 2011;10(1):225.

For peer review only - http://bmjopen.bmj.com/site/about/guidelines.xhtml

26. Farnsworth SK, Böse K, Fajobi O, et al. Community engagement to enhance child survival and early development in low-and middle-income countries: an evidence review. *Journal of health communication.* 2014;19(sup1):67-88.

27. Musa BM, Iliyasu Z, Yusuf SM, Uloko AE. Systematic review and metanalysis on community based interventions in tuberculosis care in developing countries. *Nigerian journal of medicine: journal of the National Association of Resident Doctors of Nigeria.* 2014;23(2):103-117.

28. Semrau M, Lempp H, Keynejad R, et al. Service user and caregiver involvement in mental health system strengthening in low- and middle-income countries: systematic review. *BMC Health Serv Res.* Mar 1 2016;16:79.

29. Lempp H, Abayneh S, Gurung D, et al. Service user and caregiver involvement in mental health system strengthening in low- and middle-income countries: a cross-country qualitative study. *Epidemiol Psychiatr Sci.* Feb 2018;27(1):29-39.

30. Ocloo J, Matthews R. From tokenism to empowerment: progressing patient and public involvement in healthcare improvement. *BMJ Qual Saf.* Aug 2016;25(8):626-632.

31. Harwood S. Handbook of Action Research: Participative Inquiry & Practice. *American Planning Association. Journal of the American Planning Association.* 2003;69(3):332.

32. Baum F, MacDougall C, Smith D. Participatory action research. *Journal of epidemiology and community health.* 2006;60(10):854.

33. Chevalier JM BD. Participatory action research: theory and methods for engaged inquiry. 2013(Routledge).

34. Nix E, Paulose J, Shrubsole C, et al. Participatory Action Research as a Framework for Transdisciplinary Collaboration: A Pilot Study on Healthy, Sustainable, Low-Income Housing in Delhi, India. *Glob Chall.* Apr 2019;3(4):1800054.

35. O'Reilly-de Brún M, de Brún T, O'Donnell CA, et al. Material practices for meaningful engagement: An analysis of participatory learning and action research techniques for data generation and analysis in a health research partnership. *Health Expect.* Feb 2018;21(1):159-170.

36. Brunton G, Thomas J, O'Mara-Eves A, Jamal F, Oliver S, Kavanagh J. Narratives of community engagement: a systematic review-derived conceptual framework for public health interventions. *BMC Public Health.* 2017;17(1):1-15.

37. Stringer ET. *Action research*. 4th ed. California: SAGE Publications; 2014.

38. Crane P, O'Regan M. *On PAR: Using participatory action research to improve early intervention*: Department of Families, Housing, Community Services and Indigenous Affairs …; 2010.

39. Kemmis S, McTaggart R, Nixon R. Introducing critical participatory action research. *The action research planner*. Springer; 2014:1-31.

For peer review only - http://bmjopen.bmj.com/site/about/guidelines.xhtml

40. Flicker S, Guta A, Larkin J, et al. Survey design from the ground up: Collaboratively creating the Toronto Teen Survey. *Health Promotion Practice.* 2010;11(1):112-122.

41. Viswanathan M, Ammerman A, Eng E, et al. Community-based participatory research: Assessing the evidence: Summary. *AHRQ evidence report summaries*: Agency for Healthcare Research and Quality (US); 2004.

42. Titchen A, Binnie A. Action research: a strategy for theory generation and testing. *International Journal of Nursing Studies.* 1994;31(1):1-12.

43. Hong QN, Pluye P, Bujold M, Wassef M. Convergent and sequential synthesis designs: implications for conducting and reporting systematic reviews of qualitative and quantitative evidence. *Systematic reviews.* 2017;6(1):61.

44. De Savigny D, Blanchet K, Adam T. *Applied systems thinking for health systems research: a methodological handbook*: McGraw-Hill Education; 2017.

45. Somesh K. Methods for community participation: a complete guide for practitioners. *Methods for community participation: a complete guide for practitioners.* 2002.

46. Gale NK, Heath G, Cameron E, Rashid S, Redwood S. Using the framework method for the analysis of qualitative data in multi-disciplinary health research. *BMC medical research methodology.* 2013;13(1):1-8.

47. McNiff J. *Writing and doing action research*: Sage; 2014.

48. Suter B. Validity in Qualitative Research on Personal Relationships. *Kentucky Journal of Communication.* 2009;28(2).

49. Rasmussen K. *Encyclopedia of measurement and statistics.* Vol 1: Sage; 2007.

50. Reason P, Bradbury H. *Handbook of action research: Participative inquiry and practice*: Sage; 2001.

51. Sandelowski M. The problem of rigor in qualitative research. *Advances in nursing science.* 1986.

52. Malterud K. Qualitative research: standards, challenges, and guidelines. *The lancet.* 2001;358(9280):483-488.

53. Graneheim UH, Lundman B. Qualitative content analysis in nursing research: concepts, procedures and measures to achieve trustworthiness. *Nurse education today.* 2004;24(2):105-112.

54. Pope C, Ziedland S, Mays N. Qualitative research in health care: Analysing qualitative data. 320. *BMJ.* 2000;8(320):7227.

55. Damianakis T, Woodford MR. Qualitative research with small connected communities: Generating new knowledge while upholding research ethics. *Qualitative health research.* 2012;22(5):708-718.

For peer review only

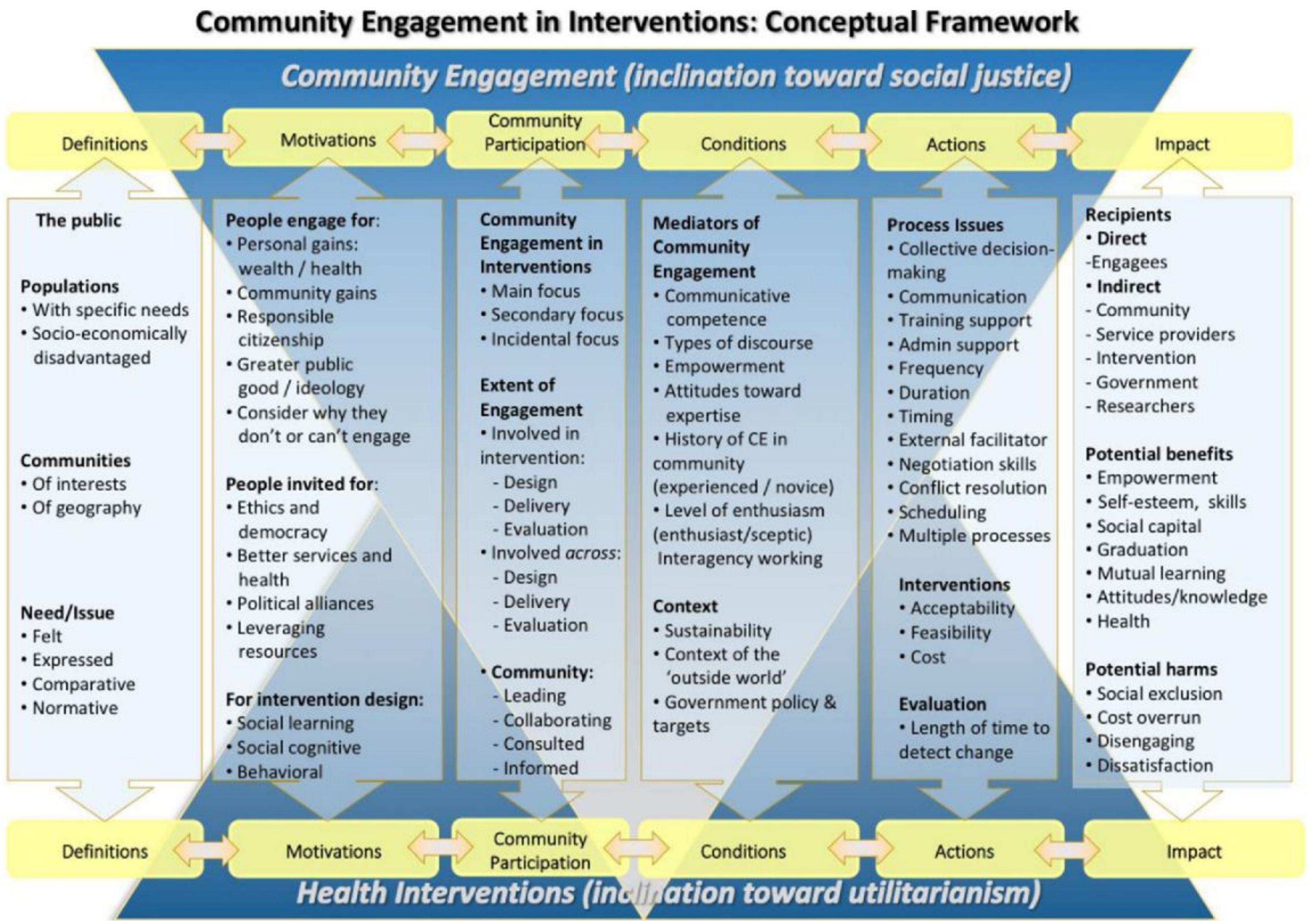

Figure 1: A conceptual framework for community engagement in interventions Brunton et al. [36].

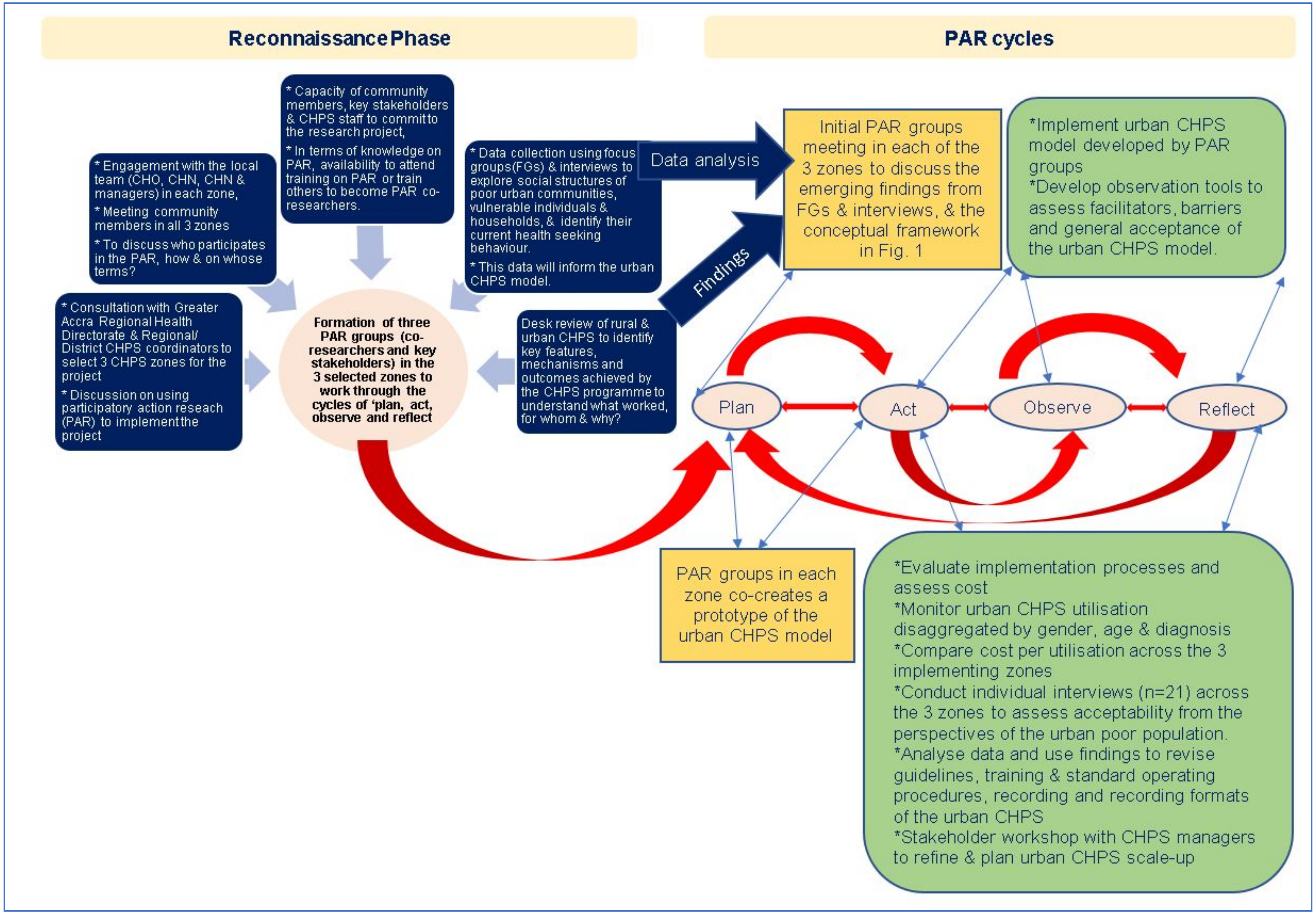

Figure 2: Proposed PAR activities and cycles for this study

BMJ Open

Adapting the community-based health planning and services (CHPS) to engage urban poor communities in Ghana: protocol for a participatory action research study.

Journal:	*BMJ Open*
Manuscript ID	bmjopen-2021-049564.R1
Article Type:	Protocol
Date Submitted by the Author:	14-Jun-2021
Complete List of Authors:	Abboah-Offei, Mary; University of York<br>Gyasi, Akosua; Ghana College of Physicians and Surgeons<br>Ayim, Andrews; Ghana College of Physicians and Surgeons<br>Ansah-Ofei, Adelaide; University of Ghana, School of Nursing and Midwifery<br>Dovlo, Delanyo; Ghana College of Physicians and Surgeons<br>Awoonor-Williams, John ; Ghana Health Service<br>Agongo, Erasmus; Ghana College of Physicians and Surgeons<br>Agyepong, Irene; Ghana Health Service<br>Elsey, Helen ; University of York,
**Primary Subject Heading**:	Public health
Secondary Subject Heading:	Health services research, Health policy, Global health
Keywords:	PUBLIC HEALTH, HEALTH SERVICES ADMINISTRATION & MANAGEMENT, PRIMARY CARE, SOCIAL MEDICINE, Public health < INFECTIOUS DISEASES

SCHOLARONE™
Manuscripts

I, the Submitting Author has the right to grant and does grant on behalf of all authors of the Work (as defined in the below author licence), an exclusive licence and/or a non-exclusive licence for contributions from authors who are: i) UK Crown employees; ii) where BMJ has agreed a CC-BY licence shall apply, and/or iii) in accordance with the terms applicable for US Federal Government officers or employees acting as part of their official duties; on a worldwide, perpetual, irrevocable, royalty-free basis to BMJ Publishing Group Ltd ("BMJ") its licensees and where the relevant Journal is co-owned by BMJ to the co-owners of the Journal, to publish the Work in this journal and any other BMJ products and to exploit all rights, as set out in our licence.

The Submitting Author accepts and understands that any supply made under these terms is made by BMJ to the Submitting Author unless you are acting as an employee on behalf of your employer or a postgraduate student of an affiliated institution which is paying any applicable article publishing charge ("APC") for Open Access articles. Where the Submitting Author wishes to make the Work available on an Open Access basis (and intends to pay the relevant APC), the terms of reuse of such Open Access shall be governed by a Creative Commons licence – details of these licences and which Creative Commons licence will apply to this Work are set out in our licence referred to above.

Other than as permitted in any relevant BMJ Author's Self Archiving Policies, I confirm this Work has not been accepted for publication elsewhere, is not being considered for publication elsewhere and does not duplicate material already published. I confirm all authors consent to publication of this Work and authorise the granting of this licence.

For peer review only

Adapting the community-based health planning and services (CHPS) to engage urban poor communities in Ghana: protocol for a participatory action research study.

Abboah-Offei, M[1]., Gyasi, A.[2], Ayim, A.[3], Ansah-Ofei, A. M.[4], Dovlo, D.[5], Awoonor-Williams J. K.[6], Agongo E. E. A[7], Agyepong, I. A.[8] and Elsey, H.[9]

Author details:

1. Dr Abboah-Offei, Mary; Department of Health Sciences, University of York Heslington, York Y10 5DD email: mary.abboah-offei@york.ac.uk
2. Dr Gyasi, Akosua; Ghana College of Physicians and Surgeons, Public Health Faculty P.O. Box MB 429, Ministries, Accra, Ghana email: akosua.gyasi@yahoo.com
3. Dr Ayim, Andrews; Address: Ghana College of Physicians and Surgeons, Public Health Faculty P.O. Box MB 429, Ministries, Accra, Ghana Mobile: +233244230466 email: andyayim@yahoo.co.uk
4. Dr Ansah-Ofei, Adelaide Maria; School of Nursing and Midwifery, University of Ghana, Legon Tel. 0244653065/0204653065 email: adelaideofei@gmail.com
5. Dr Dovlo, Delanyo; Ghana College of Physicians and Surgeons, Public Health Faculty P.O. Box MB 429, Ministries, Accra, Ghana email: dovlod@gmail.com
6. Dr Awoonor-William, John Koku; Director, Policy Planning Monitoring and Evaluation, Ghana Health Service Private Mail Bag, Ministries, Accra, Ghana Tel: +233-208161394 +233 244564120 email: kawoonor@gmail.com
7. Dr Agongo, Erasmus Emmanuel Akurugu; Ghana College of Physicians and Surgeons, Public Health Faculty P.O. Box MB 429, Ministries, Accra, Ghana email: erasmusagongo@yahoo.co.uk
8. Professor Agyepong, Irene Akua; Ghana Health Service Research and Development Division Dodowa Health Research Center, P.O. Box DD 1, Dodowa. Tel. 233-244 862 665 /233-208 133 850; email: irene.agyepong@gmail.com.
9. Dr Elsey, Helen; Department of Health Sciences, University of York Heslington, York Y10 5DD Tel: 0790 4681241 email: helen.elsey@york.ac.uk

For peer review only

Corresponding author:

Mary Abboah-Offei

Department of Health Sciences

University of York

Heslington, York YO10 5DD

E-mail: mary.abboah-offei@york.ac.uk

Tel. 07500045742

For peer review only

Abstract

Introduction: With rapid urbanisation in low- and middle-income countries, health systems are struggling to meet the needs of their growing populations. Community-based Health Planning and Services (CHPS) in Ghana have been effective in improving maternal and child health in rural areas however, implementation in urban areas has proved challenging. This study aims to engage key stakeholders in urban communities to understand how the CHPS model can be adapted to reach urban poor communities.

Methods and analysis: A Participatory Action Research (PAR) will be used to develop an urban CHPS model with stakeholders in three selected CHPS zones (a. Old Fadama (Yam and Onion Market community), b. Adedenkpo and c. Adotrom 2) representing three categories of poor urban neighbourhoods in Accra, Ghana. Two phases will be implemented: Phase one ('Reconnaissance phase) will engage and establish PAR research groups in the selected zones, conduct focus groups and individual interviews with urban residents, households vulnerable to ill-health and CHPS staff and key stakeholders. A desk review of preceding efforts to implement CHPS will be conducted to understand what worked (or not), how and why. Findings from Phase one will be used to inform and co-create an urban CHPS model in Phase two, where PAR groups will be involved in multiple recurrent stages (cycles) of community-based planning, observation, action and reflection to develop and refine the urban CHPS model. Data will be managed using NVivo software and coded using the domains of community engagement as a framework to understand community assets and potential for engagement.

Ethics and dissemination: This study has been approved by the University of York's Health Sciences Research Governance Committee and the Ghana Health Service Ethics Review Committee. The results of this study will guide the scale-up of CHPS across urban areas in Ghana, which will be disseminated through journal publications, community and government stakeholder workshops, policy briefs and social media content. This study is also funded by the Medical Research Council, United Kingdom.

Keywords: Ghana, CHPS, Urban communities, Community engagement, Participatory Action Research

Word count: 3, 951

Article summary:

For peer review only - http://bmjopen.bmj.com/site/about/guidelines.xhtml

Strengths and limitations of this study

- Close engagement with Ghana Health Service and communities throughout the study will enable the development of an urban CHPS model that can be delivered sustainably within the current health system.
- Using PAR will enable increased engagement and the collaboration with research participants and stakeholders.
- The mixed methods used within the PAR approach in three different urban poor neighbourhoods will provide in-depth understanding of the health needs of vulnerable urban residents.
- PAR is time intensive and will require prolonged engagement with the research setting and stakeholders.
- Given the level of engagement involving the study, the COVID-19 pandemic presents a major risk to our ability to implement the study as planned.

For peer review only

Introduction

Sub Saharan Africa is urbanising fast; with over half of the continent's population predicted to live in urban areas by 2035[1]. Governments, at national and city level, face multiple challenges in addressing the growing health needs of their expanding populations[2]. Despite outdated notions of an 'urban advantage', proximity to healthcare does not equate to access to free, high quality healthcare services or health promoting interventions for the urban poor[3]. This often result in worse outcomes than their rural counterparts and better-off urban residents[4]. Ghana is one of the most urbanised countries in sub-Saharan Africa with 56.7% of the population estimated to be living in urban areas in 2019[5]. These rapidly expanding cities and towns are characterized by slum and peri-urban communities with poor infrastructure, over-crowded and unsanitary conditions, which increases the risk for both communicable and non-communicable diseases, resulting in inequalities, poverty and marginalization[2,6]. With public services struggling to reach urban poor communities, people turn to a range of predominantly private clinics and pharmacies, however, with limited uptake of health insurance by the poorest [6] their access to quality and affordable health-care services and health promoting activities are severely limited. This has impacted on infant and child mortality which are five times higher in poor urban communities compared to the general urban population [6]. Ensuring appropriate, quality service delivery to households will improve timely, suitable care and health promotion activities and reduce vulnerability to expensive and inappropriate care delivery through a plethora of unregulated providers.

Ghana's three-tier district health system has at its foundation the Community-based Health Planning and Services (CHPS) programme, which has been successfully delivering universal access to health promotion, prevention and basic curative care in rural districts using community-based nurses known as Community Health Officers (CHOs) and volunteers [7-16]. This has led to a reduction in childhood mortality by a third [17] and decline in total fertility by one birth [18]. However, despite government policy to scale-up CHPS nationally, these benefits do not currently extend to the urban population [19,20]. CHPS implementation in urban areas of Ghana has been limited to a few pilot districts [7] and there are calls for more research to inform an urban CHPS model [8]. Evidence from CHPS piloted in some urban areas have revealed a need for greater range of services including improved approaches to the delivery of the Integrated Management of Childhood Illnesses (IMCI); sustained and expanded engagement of communities and volunteering programmes; improved motivation and skills training for staff and opportunities for career progression [20]. Limited registration with Ghana's National Health Insurance Scheme (NHIS) further undermines access to health services in urban areas [21]. NHIS was created in 2003 with the ideal of being 'pro-poor', however, recent studies have shown only 17% of the poor are insured compared to 44% among rich households [22]. Although those classified as extremely poor automatically qualify for a free NHIS card [23], the extent to which this is taken-up in poor urban areas is unknown.

For peer review only - http://bmjopen.bmj.com/site/about/guidelines.xhtml

Developing appropriate and sustainable system-wide solutions in the urban context is vital if urban CHPS is to move beyond a few pilot areas.

Community engagement is a central pillar of CHPS with six milestones of implementation [8], however in many poor urban areas social structures and cohesion look very different from those found in rural areas. Urban poor neighbourhoods are frequently characterised transient migrant populations, with both men and women working long hours with reduced support from extended family and multiple stresses of urban living [24]. Studies have identified particular challenges in engaging urban communities in health programmes and activities [25-27]. There are many examples of community engagement approaches, particularly in rural areas, such as the social accountability approach which has been described by the World Health Organisation (WHO) as a method of community engagement that brings relevant perspectives together in conversations where everyone is an equal partner [28]. Other key examples of community engagements that have led to successful roll out of community clinics targeting urban poor populations are those of the Mohalla and Basthi Dawakhana clinics in India [29,30]. Understanding how best to engage urban poor households, particularly the most vulnerable women and children is vital for effective adaptation and scale up of CHPS within urban areas. Methods for community engagement in decisions that affect their lives have been criticized for the lack of participatory approaches and inability to move beyond tokenistic mode of participation [31-33].

One approach to addressing these criticisms is Participatory Action Research (PAR) [34,35], which will help to identify and specify system adaptations required to effectively implement CHPS sustainably to reach the urban poor. The PAR approach will enable meaningful engagement with stakeholders, communities and vulnerable households in identifying social structures, health needs and other areas of concern through collaboration and capacity building to address these issues holistically [36-38]. In order to enhance a better understanding of engagement within health systems, this study has adopted the conceptual framework derived from a systematic review of public health interventions (see Figure 1)[39]. This framework provides clarity on aspects of engagement from a community and a health system perspective, and has the advantage of being an empirically driven model utilising findings from both qualitative and quantitative studies[39]. Although studies included in this review were predominantly from high-income countries, the insights show similarities to reviews of engagement in low and middle-income countries (LMICs) [25-27].

Therefore this study aims to engage CHPS team and managers, volunteers, community members and key stakeholders to understand their social structures, health needs, health seeking and health insurance behaviour, and to identify and specify system adaptations required to effectively implement CHPS sustainably and at-scale to reach the urban poor in Ghana.

For peer review only - http://bmjopen.bmj.com/site/about/guidelines.xhtml

Insert Figure 1: A conceptual framework for community engagement in interventions Brunton et al. [39].

Study objectives

1. To explore social structures of poor urban communities, and vulnerable individuals and households, including the uninsured, and identify their current health seeking behaviour, using this information to adapt the CHPS model.
2. To review and critically analyse preceding efforts to implement rural and urban CHPS to understand what worked (or not), how and why.
3. To design with urban communities, CHPS community teams and health system managers, an adaptation of the current CHPS programme and system to ensure urban relevance.
4. To evaluate early implementation processes, costs, and acceptability from the perspective of urban populations who are marginalized and vulnerable such as women and children and frontline health workers, volunteers and health systems managers and identify the pathway for scale up.
5. To make recommendations on contextually appropriate modifications to the CHPS and NHIS policy, programme and implementation arrangements for urban localities as well as approaches to scale up

Methods

Study design

The study uses both quantitative and qualitative methods within the overall approach of participatory action research (PAR). PAR is a rigorous and systematic approach to enquiry, which allows researchers and stakeholders to explore and discover effective solutions to life problems [40]. PAR gives stakeholders the opportunity to be involved with multiple recurrent stages (cycles) of community-based planning, action, observation and reflection[41] with each cycle following on from and influencing subsequent cycles [40,42]. The use of PAR in the community is beneficial in increasing engagement and the collaborative nature of the study [43,44]. We chose PAR as an appropriate methodology for this study as it will enable us to try out different approaches to engagement in a range of urban poor settings and reflect on the experience in collaboration with CHOs, volunteers and community members.

In addition, given that community engagement is a key component of CHPS, the alignment of PAR to community engagement will help to strengthen CHPS staff skills in participation approaches,

For peer review only - http://bmjopen.bmj.com/site/about/guidelines.xhtml

whilst simultaneously strengthening engagement bonds with poor urban communities. This level of engagement we hope will increase community ownership of the urban CHPS model, supporting scale-up and sustainability. For example, we will clarify, from the perspective of communities and CHPS, which population groups and communities should be targeted and understand the motivations, both from communities and CHPS, for their inclusion. This will include understanding the motivations for registering (or not) for NHIS and facilitating those excluded from NHIS to identify ways to encourage registration among these groups. We will also explore the extent of participation, the mediators and context for engagement. Our PAR cycles will allow us to consciously address process issues and document the successes for different approaches to process. We will explore the impact of community engagement on participants, the wider community, within the CHPS teams and the health system (both potential benefits and harms). This study will be implemented in two phases: Phase one will address study objectives 1 to 2, which will constitute 'Reconnaissance phase' in PAR (see Figure 2); and Phase 2 will address study objectives 3 to 5 and will comprise a number of PAR cycles (see Figure 3).

Setting

We purposively selected three poor urban CHPS zones: (a) Old Fadama (Yam and Onion Market community), (b) Adedenkpo and (c) Adotrom 2) that do not currently have functional CHPS programme (i.e. a designated CHPS zone that is lacking in facilities, staff or targeted outreach services), and with differing characteristics, to allow for transferability across urban areas of differing social characteristics. A CHPS zone includes about 5,000 to 10,000 population assigned to a Community Health Team led by a CHO. CHPS zones are linked to primary health clinics/maternity homes as the next level of care, and then to District/Municipal Hospital and Health Management Team. This three-tier system allows for referral as well as supervision and monitoring from the community level. The three selected zones will include: (a) an informal settlement of predominantly first-generation migrants; (b) a mixed poor/better-off neighbourhood; and (c) a long-established neighbourhood of several generations. We will also select different levels of CHPS functionality (e.g. with facilities and staff but no volunteers or outreach, or with no facilities or staff in the demarcated zone but with support from neighbouring zones).

STUDY PHASE 1

Reconnaissance phase

The 'Reconnaissance Phase' [45] is described in the action research literature as an observational phase to gain insight into the problem and develop a theorised-account to inform action[45]. The information generated in the reconnaissance phase will inform and enable the establishment of groups of co-researchers in each of the three CHPS zones. The findings of the reconnaissance phase will also inform the CHPS models to be tried in the first PAR cycle in each CHPS zone. PAR groups will be made up of key stakeholders, or co-researchers, affected by the health and

For peer review only - http://bmjopen.bmj.com/site/about/guidelines.xhtml

access problems experienced in their neighbourhoods. The co-research groups will include community members, CHPS staff, volunteers and mangers, policy makers and representatives from the regional health directorate. The reconnaissance phase enables theory building within action-research[45], emerging theories will be structured according to the theoretical framework in Figure 1 [39]. We will also be mindful of emerging aspects not reflected in the framework; this is particularly important given the limited LMIC-based studies that have informed this framework.

This phase will focus on consultations with Greater Accra Regional Health Directorate and Regional/ District CHPS coordinators and the Accra Metropolitan Assembly to select 3 CHPS zones for the study; engagement and transect walks with the local team (CHPS team & managers, community members and other key stakeholders) in each zone; checking and building capacity of community members, key stakeholders & CHPS staff to commit to the study in terms of knowledge on PAR, availability to attend training on PAR or train others to become PAR co-researchers. This phase will address research objectives 1 and 2, enabling us to find a shared concern among key stakeholders and community members in their respective CHPS zones to begin to identify solutions from the perspective of those most affected [42], particularly community members vulnerable to ill-health and poor access to health services and CHPS staff and volunteers (see Figure 2 for activities in the Reconnaissance Phase).

Insert Figure 2: PAR activities in Reconnaissance Phase

For objective 2, we will conduct a mixed methods review using a results-based convergent design[46], where the quantitative and qualitative findings are synthesised separately and then brought together in a final synthesis. This will allow us to synthesise quantitative results from included studies on the outcomes of CHPS and findings from qualitative, mixed-methods or quantitative studies on the mechanisms (e.g. health system, participant or contextual factors) that may influence effectiveness. We will include any study evaluating the CHPS programme in urban or rural Ghana and will particularly look to identify facilitators and barriers to success in urban contexts. We will also establish the groups of co-researchers in each CHPS zones, share the findings form the reconnaissance work, and facilitate discussions on adaptations of the CHPS model to respond to community needs.

Recruitment and data collection in Phase 1

A purposive sample of key health providers and community stakeholders will be recruited from the three selected zones for an initial focus group (FG) discussion. Due to power play and political factions that exist in selected communities, stakeholder groups (policy makers, regional health directorate, CHPS staff and community members) will be engaged separately in focus groups and

For peer review only - http://bmjopen.bmj.com/site/about/guidelines.xhtml

individual interviews prior to engaging all stakeholder groups together in the PAR groups. This will allow for the exploration of perspectives and issues within respective communities, where community members can talk freely without fear of intimidation from other stakeholders. Then during the PAR groups meeting, issues raised in the focus groups and interviews will be brought to the table where everyone will discuss in an environment of respect and equity [28]. Two focus groups (FGs) each will be conducted in each zone using techniques such as 'social-mapping' to facilitate discussion on social structures and health seeking behaviour, and 'chapati diagrams' [47] to identify health providers and their relative importance (each FG will comprise of 8 to 10 participants, and a total of n=6 FGs will be held). Local gatekeepers from the FGs will help researchers to identify particularly vulnerable household (e.g. with under-5 children, female-headed, elderly or chronically ill or disabled) for individual interviews to explore the challenges they face in registering for insurance, keeping healthy and seeking care. Eight individual interviews will be conducted in each zone making a total of n=24 interviews informing 'definitions' and 'motivations' within the framework [39]. The sequential follow-up of individual interviews is aimed at gaining a deeper understanding of the internal and external issues and concerns surrounding the implementation and use of CHPS services.

Data will be collected on baseline costs and utilisation, disaggregated by gender and age, of any elements of from existing Urban CHPS pilot. The findings of the mixed-methods systematic review of the CHPS programme in rural and urban areas will be used to inform key informant individual interviews with CHPS staff and volunteers (n=10) and community members (n=10) in the three selected zones. These interviews will explore all six aspects of the framework [39] in Figure 1.

Data analysis for Phase 1

All interviews and focus groups will be audio recorded and transcribed as soon after data collection as possible. Transcripts will be translated into English. Data collected will be reviewed and coded using the Brenton et al domains of community engagement as a framework to understand community assets and potential for engagement. The data collected in phase one will also enable identification of the needs, gaps, weaknesses and opportunities within the three selected zones relating to CHPS, staff and volunteers, community members and other relevant stakeholders. We will follow the seven stages within Framework Approach as described by Gale et al[48]. Data will be managed using NVivo software. This analysis will inform the initial CHPS models considered by the co-researchers in each zone.

Throughout the study, the research team will keep a comprehensive reflective research journal, which will be used to catalogue the progress, obstacles and successes of the PAR process. This journal will be kept to acknowledge the research team's experience of PAR in the urban context, analysis and interpretation [49,50]. The journal will also act as a component of the audit trail for the

For peer review only - http://bmjopen.bmj.com/site/about/guidelines.xhtml

study [51]. Reflective journals also increases external validity by making subjective processes transparent [50]. During this phase the CHPS staff, volunteers and researchers will receive training and support on participatory methods and the principles underpinning PAR : participation: “Action research is only possible with, for and by persons and communities, ideally involving all stakeholders both in the questioning and sense making that informs the research, and in the action which is its focus"[52]; and produce practical knowledge, and to do that draws on representational, relational and reflective knowledge and is for a worthwhile purpose[52].

STUDY PHASE 2: PAR Cycles

During PAR cycles in study phase 2, the three groups of co-researchers will work through the cycles of ‘plan, act, observe, reflect’ [34] in their respective CHPS zones, developing the model. The PAR co-researchers (PAR groups) will work through PAR cycles to co-create the urban CHPS model with stakeholders, implement and evaluate the processes, cost and acceptability of this model from the perspectives of the urban poor population. To initiate this process, stakeholder meetings will be held with the PAR groups in each of the three zones to co-create an urban CHPS model drawing on the findings from Phase 1. The emerging findings from Phase 1 in relation to the conceptual framework[39] adopted (see Figure 1) will be discussed with the PAR groups to trigger and inform the design of the prototype urban CHPS model. Different design options will be costed to enable consideration of sustainability and scale-up at this early stage. We will also work closely with Ghana Health Service and the CHPS programme, taking an embedded research approach[53] to ensure that issues of sustainability and scale-up are central to the design.

Following from the workshop with the PAR groups from the three zones, the co-researchers in each zone will begin the PAR cycles. The PAR groups will be facilitated by public health professionals in training (residents) from the Ghana College of Physicians and Surgeons (GCPS), who will also document the decisions taken by the PAR groups and support them to identify the most effective methods for assessing whether the adaptations to the CHPS model work in practice in increasing access, feasibility and appropriateness of CHPS in their community. This observation stage will include use of routine health information data to understand patterns of utilisation of CHPS services disaggregated by gender, age and diagnosis (variables routinely available in the District Health Information Management Systems (DHIMS)). The PAR groups will monitor utilisation data on an on-going basis identifying those excluded and responding by reshaping aspects of the model in subsequent PAR cycles (see Figure 3 for PAR cycles and activities in Phase 2).

Insert Figure 3: Proposed PAR cycles and activities

For peer review only - http://bmjopen.bmj.com/site/about/guidelines.xhtml

Data collection in Phase 2

The co-researchers and public health residents will use qualitative methods including observation, interviews and focus groups as appropriate to understand issues of acceptability, feasibility and access in more depth and from the perspective of those most likely to struggle in accessing health care and improving health. Actual costs of delivering the model from the health service perspective in the different urban zones will be collected. This will include costs of staff and volunteer training, time and salary or expenses, transportation, materials and equipment, supervision. The utilisation data will be used to estimate the cost per patient in each of the three zones. Emerging findings from the co-researchers in the three zones will be discussed with GHS and CHPS programme managers over the 12 months period of implementation of the PAR cycles. At the end of the 12 months, a workshop will be held with all stakeholder groups (CHPS managers & staff, provincial and national health system stakeholders, policy makers and community members to assess the findings from the selected zones in order to plan the next steps in implementation and scale-up, if appropriate. We will develop guidelines, training and standard operating procedures, recording and reporting formats for the urban CHPS model based on study findings (see figure 4 for all study activities in Phases 1 & 2).

Insert Figure 4: Proposed PAR activities and cycles for the whole study

For peer review only

For peer review only - http://bmjopen.bmj.com/site/about/guidelines.xhtml

Patient and public involvement

Community members from the selected CHPS zones will be involved throughout the study. The community leaders and the CHPS managers in these selected communities will assist in negotiating access to the community. This will include local recruitment of PAR co-researchers and hosting of series of community engagement meetings with PAR groups where the prototype urban CHPS model design will be discussed before the results are disseminated.

Rigour

Appropriate measures will be implemented to increase the rigour of this study. Data will be collected and coded by the research team and discussions held regularly with stakeholders to reduce bias [54]. Sources of potential bias that could influence the processes of data collection and analysis due to existing networks and connections will be acknowledged and recorded. This level of documentation will increase confirmability by providing an audit trail, which will allow observers to confirm the veracity of the study [40,55]. Prolonged engagement with the community and stakeholders will increase credibility and regular member checking of raw data, analyses and reports [40]. Transferability will be provided through detailed descriptions of the contextual data and activities of the study, through immersion, reflective journaling and detailed documentation, this will allow other researchers to analyse the situation and study outcomes based on context [55,56].

The coding and themes will be analysed by at least 2 to 3 members of the research team to enhance credibility[57]. This will involve a reflective practice whereby the team leader will first code the data, and then these codes will be discussed by the research team and further refined to ensure the codes fit with the framework and any emerging themes reflect the dataset. This process will enhance dependability and inter-coder reliability[57]. The research team will be involved in the development of all interview guides and further refinement of the guide will occur as a team.

Ethics approval

This study has been approved by the University of York's Health Sciences Research Governance Committee (HSRGC/2020/409/E) and the Ghana Health Service Ethics Review Committee (GHS-ERC 003/10/20). Given the community participatory nature of this study, there are ethical considerations in relation to protecting the anonymity of participants and confidentiality of data, particularly regarding interviews and focus groups. The connected nature of community members will be acknowledged in consent forms, ground-rules of confidentiality will be agreed in all group discussions, care will be taken in analysis, and presentation of data in ensuring that participant confidentiality is protected. Data that may overtly identify participants will be excluded [58]. Consent will be required from all participants prior to their participation in the study.

Capacity building and dissemination

For peer review only - http://bmjopen.bmj.com/site/about/guidelines.xhtml

This study aims to develop a centre of excellence for training and build capacity for Urban CHPS, which will develop and deliver training for CHPS urban teams on clinical and participatory techniques and processes. Under guidance of the local research team in Ghana, public health registrars training under the GCPS will be seconded to the centre evaluating, developing and supporting CHPS throughout scale-up. The PAR process will build capacity of CHPS workers in participatory approaches and use of DHIMS data to develop strategies to reach those who may struggle to access CHPS services.

The results of this study will guide the scale-up of CHPS across urban areas in Ghana providing detailed information on all components, costs and impact on utilisation, with outputs such as training materials, operational guidelines and policy revisions. This will lay the foundation for a community-driven model that fits sustainably within the GHS. The centre of excellence will facilitate further evaluation and sharing of good practice. Beyond Ghana, this study, and the planned follow-on evaluation of scale-up will provide much needed evidence and insight into how to engage communities in urban areas so their needs are addressed appropriately by the health system. Policymakers, practitioners and researchers involved in urban health across LMICs are grappling with these issues and searching for solutions to improve the health of the poorest urban residents.

For peer review only

Acknowledgements

We acknowledge all authors for their contribution to this protocol.
We also acknowledge the University of York, Ghana College of Physicians and Surgeons and the Ghana Health Service for supporting this study. Finally, we acknowledge the Medical Research Council for funding this study.

Author contributions

AEEA, EH, AIA, KJA-W developed the concept for the study and successful sought funds from Medical Research Council to conduct the study. MA-O developed and drafted the initial manuscript. GA, AA, AAM, and DD reviewed the initial draft of the manuscript and added to the methodology, plans for analysis and dissemination. All authors reviewed and final draft of the manuscript and approving the final version.

Funding statement

The Medical Research Council grant number MR/T022787/1 support this work.

Competing interest statement

For peer review only - http://bmjopen.bmj.com/site/about/guidelines.xhtml

None

For peer review only

For peer review only - http://bmjopen.bmj.com/site/about/guidelines.xhtml

References

1. UN DoE, Social Affairs PD. World Urbanization Prospects: The 2018 Revision (ST/ESA/SER. A/420): United Nations New York; 2019.
2. Elsey H, Agyepong I, Huque R, et al. Rethinking health systems in the context of urbanisation: challenges from four rapidly urbanising low-income and middle-income countries. *BMJ Glob Health.* 2019;4(3):e001501.
3. Shawar YR, Crane LG. Generating global political priority for urban health: the role of the urban health epistemic community. *Health policy and planning.* 2017;32(8):1161-1173.
4. Mberu BU, Haregu TN, Kyobutungi C, Ezeh AC. Health and health-related indicators in slum, rural, and urban communities: a comparative analysis. *Global health action.* 2016;9(1):33163.
5. UNData. Ghana. 2019; https://data.un.org/en/iso/gh.html. Accessed October 14, 2020.
6. Taylor P, Boussen CR, Awunyo-Akaba J, Nelson J. Urban Health Assessment. *Prepared for the USAID Mission to Ghana, Activity Report.* 2002;114.
7. Johnson FA, Frempong-Ainguah F, Matthews Z, et al. Evaluating the impact of the community-based health planning and services initiative on uptake of skilled birth care in Ghana. *PLoS One.* 2015;10(3):e0120556.
8. Nyonator FK, Awoonor-Williams JK, Phillips JF, Jones TC, Miller RA. The Ghana community-based health planning and services initiative for scaling up service delivery innovation. *Health policy and planning.* 2005;20(1):25-34.
9. Awoonor-Williams JK, Sory EK, Nyonator FK, Phillips JF, Wang C, Schmitt ML. Lessons learned from scaling up a community-based health program in the Upper East Region of northern Ghana. *Global Health: Science and Practice.* 2013;1(1):117-133.
10. Awoonor-Williams JK, Bawah AA, Nyonator FK, et al. The Ghana essential health interventions program: a plausibility trial of the impact of health systems strengthening on maternal & child survival. *BMC health services research.* 2013;13(S2):S3.
11. Awoonor-Williams JK, Appiah-Denkyira E. Bridging the intervention-implementation gap in primary health care delivery: the critical role of integrated implementation research. *BMC Health Services Research.* 2017;17(3):91-94.
12. Nyonator FK, Akosa AB, Awoonor-Williams JK, Phillips JF, Jones TC. Scaling up experimental project success with the Community-based Health Planning and Services initiative in Ghana. *Scaling up health service delivery: from pilot innovations to policies and programmes. Geneva: WHO.* 2007:89-112.
13. Awoonor-Williams J, Feinglass E, Tobey R, Vaughan-Smith M, Nyonator F, Jones T. The impact of a replication project on safe motherhood and family planning behavior in Nkwanta District of Ghana. *Studies in Family Planning.* 2004;35:161-177.
14. Ngom P, Debpuur C, Akweongo P, Adongo P, Binka FN. Gate-keeping and women's health seeking behaviour in Navrongo, northern Ghana. *African journal of reproductive health.* 2003:17-26.
15. Binka FN, Nazzar A, Phillips JF. The Navrongo community health and family planning project. *Studies in family planning.* 1995:121-139.
16. Atinga RA, Agyepong IA, Esena RK. Ghana's community-based primary health care: why women and children are 'disadvantaged'by its implementation. *Social Science & Medicine.* 2018;201:27-34.
17. Pence B, Nyarko P, Binka FN, Phillips JF, Debpuur C. The impact of the Navrongo Community Health and Family Planning Project on child mortality, 1993–2000. Paper presented at: Global Conference of the International Union for the Scientific Study of Population, Salvador, Brazil, August2001.
18. Debpuur C, Phillips JF, Jackson EF, Nazzar A, Ngom P, Binka FN. The impact of the Navrongo Project on contraceptive knowledge and use, reproductive preferences, and fertility. *Studies in family planning.* 2002;33(2):141-164.
19. Adongo PB, Phillips JF, Aikins M, et al. Does the design and implementation of proven innovations for delivering basic primary health care services in rural communities fit the urban setting: the case of Ghana's Community-based Health Planning and Services (CHPS). *Health research policy and systems.* 2014;12(1):16.
20. Nwameme AU, Tabong PT-N, Adongo PB. Implementing community-based health planning and services in impoverished urban communities: health workers' perspective. *BMC health services research.* 2018;18(1):1-11.
21. Kwarteng A, Akazili J, Welaga P, et al. The state of enrollment on the National Health Insurance Scheme in rural Ghana after eight years of implementation. *Int J Equity Health.* Dec 31 2019;19(1):4.
22. Kotoh AM, Aryeetey GC, Van Der Geest S. Factors that influence enrolment and retention in Ghana'National Health Insurance Scheme. *International journal of health policy and management.* 2018;7(5):443.

For peer review only - http://bmjopen.bmj.com/site/about/guidelines.xhtml

23. Handa S, Park M, Darko RO, Osei-Akoto I, Davis B, Daidone S. Livelihood empowerment against poverty program impact evaluation. *Chapel Hill: Carolina Population Center, University of North Carolina.* 2013.

24. Lyons M, Snoxell S. Creating urban social capital: some evidence from informal traders in Nairobi. *Urban Studies.* 2005;42(7):1077-1097.

25. Atkinson J-A, Vallely A, Fitzgerald L, Whittaker M, Tanner M. The architecture and effect of participation: a systematic review of community participation for communicable disease control and elimination. Implications for malaria elimination. *Malaria journal.* 2011;10(1):225.

26. Farnsworth SK, Böse K, Fajobi O, et al. Community engagement to enhance child survival and early development in low-and middle-income countries: an evidence review. *Journal of health communication.* 2014;19(sup1):67-88.

27. Musa BM, Iliyasu Z, Yusuf SM, Uloko AE. Systematic review and metanalysis on community based interventions in tuberculosis care in developing countries. *Nigerian journal of medicine: journal of the National Association of Resident Doctors of Nigeria.* 2014;23(2):103-117.

28. Organization WH. *Towards unity for health: challenges and opportunities for partnership in health development*: World Health Organization;2000.

29. Lahariya C. Mohalla Clinics of Delhi, India: Could these become platform to strengthen primary healthcare? *Journal of family medicine and primary care.* 2017;6(1):1.

30. Lahariya C. Basthi Dawakhana of Hyderabad: The first Urban Local Body led community clinics in India. *Journal of family medicine and primary care.* 2019;8(4):1301.

31. Semrau M, Lempp H, Keynejad R, et al. Service user and caregiver involvement in mental health system strengthening in low- and middle-income countries: systematic review. *BMC Health Serv Res.* Mar 1 2016;16:79.

32. Lempp H, Abayneh S, Gurung D, et al. Service user and caregiver involvement in mental health system strengthening in low- and middle-income countries: a cross-country qualitative study. *Epidemiol Psychiatr Sci.* Feb 2018;27(1):29-39.

33. Ocloo J, Matthews R. From tokenism to empowerment: progressing patient and public involvement in healthcare improvement. *BMJ Qual Saf.* Aug 2016;25(8):626-632.

34. Harwood S. Handbook of Action Research: Participative Inquiry & Practice. *American Planning Association. Journal of the American Planning Association.* 2003;69(3):332.

35. Baum F, MacDougall C, Smith D. Participatory action research. *Journal of epidemiology and community health.* 2006;60(10):854.

36. Chevalier JM BD. Participatory action research: theory and methods for engaged inquiry. 2013(Routledge).

37. Nix E, Paulose J, Shrubsole C, et al. Participatory Action Research as a Framework for Transdisciplinary Collaboration: A Pilot Study on Healthy, Sustainable, Low-Income Housing in Delhi, India. *Glob Chall.* Apr 2019;3(4):1800054.

38. O'Reilly-de Brún M, de Brún T, O'Donnell CA, et al. Material practices for meaningful engagement: An analysis of participatory learning and action research techniques for data generation and analysis in a health research partnership. *Health Expect.* Feb 2018;21(1):159-170.

39. Brunton G, Thomas J, O'Mara-Eves A, Jamal F, Oliver S, Kavanagh J. Narratives of community engagement: a systematic review-derived conceptual framework for public health interventions. *BMC Public Health.* 2017;17(1):1-15.

40. Stringer ET. *Action research*. 4th ed. California: SAGE Publications; 2014.

41. Crane P, O'Regan M. *On PAR: Using participatory action research to improve early intervention*: Department of Families, Housing, Community Services and Indigenous Affairs ...; 2010.

42. Kemmis S, McTaggart R, Nixon R. Introducing critical participatory action research. *The action research planner*: Springer; 2014:1-31.

43. Flicker S, Guta A, Larkin J, et al. Survey design from the ground up: Collaboratively creating the Toronto Teen Survey. *Health Promotion Practice.* 2010;11(1):112-122.

44. Viswanathan M, Ammerman A, Eng E, et al. Community-based participatory research: Assessing the evidence: Summary. *AHRQ evidence report summaries*: Agency for Healthcare Research and Quality (US); 2004.

45. Titchen A, Binnie A. Action research: a strategy for theory generation and testing. *International Journal of Nursing Studies.* 1994;31(1):1-12.

46. Hong QN, Pluye P, Bujold M, Wassef M. Convergent and sequential synthesis designs: implications for conducting and reporting systematic reviews of qualitative and quantitative evidence. *Systematic reviews.* 2017;6(1):61.

47. Somesh K. Methods for community participation: a complete guide for practitioners. *Methods for community participation: a complete guide for practitioners.* 2002.

48. Gale NK, Heath G, Cameron E, Rashid S, Redwood S. Using the framework method for the analysis of qualitative data in multi-disciplinary health research. *BMC medical research methodology.* 2013;13(1):1-8.

For peer review only - http://bmjopen.bmj.com/site/about/guidelines.xhtml

49. McNiff J. *Writing and doing action research*: Sage; 2014.

50. Suter B. Validity in Qualitative Research on Personal Relationships. *Kentucky Journal of Communication.* 2009;28(2).

51. Rasmussen K. *Encyclopedia of measurement and statistics.* Vol 1: Sage; 2007.

52. Reason P, Bradbury H. *Handbook of action research: Participative inquiry and practice*: Sage; 2001.

53. De Savigny D, Blanchet K, Adam T. *Applied systems thinking for health systems research: a methodological handbook*: McGraw-Hill Education; 2017.

54. Sandelowski M. The problem of rigor in qualitative research. *Advances in nursing science.* 1986.

55. Malterud K. Qualitative research: standards, challenges, and guidelines. *The lancet.* 2001;358(9280):483-488.

56. Graneheim UH, Lundman B. Qualitative content analysis in nursing research: concepts, procedures and measures to achieve trustworthiness. *Nurse education today.* 2004;24(2):105-112.

57. Pope C, Ziedland S, Mays N. Qualitative research in health care: Analysing qualitative data. 320. *BMJ.* 2000;8(320):7227.

58. Damianakis T, Woodford MR. Qualitative research with small connected communities: Generating new knowledge while upholding research ethics. *Qualitative health research.* 2012;22(5):708-718.

For peer review only

For peer review only - http://bmjopen.bmj.com/site/about/guidelines.xhtml

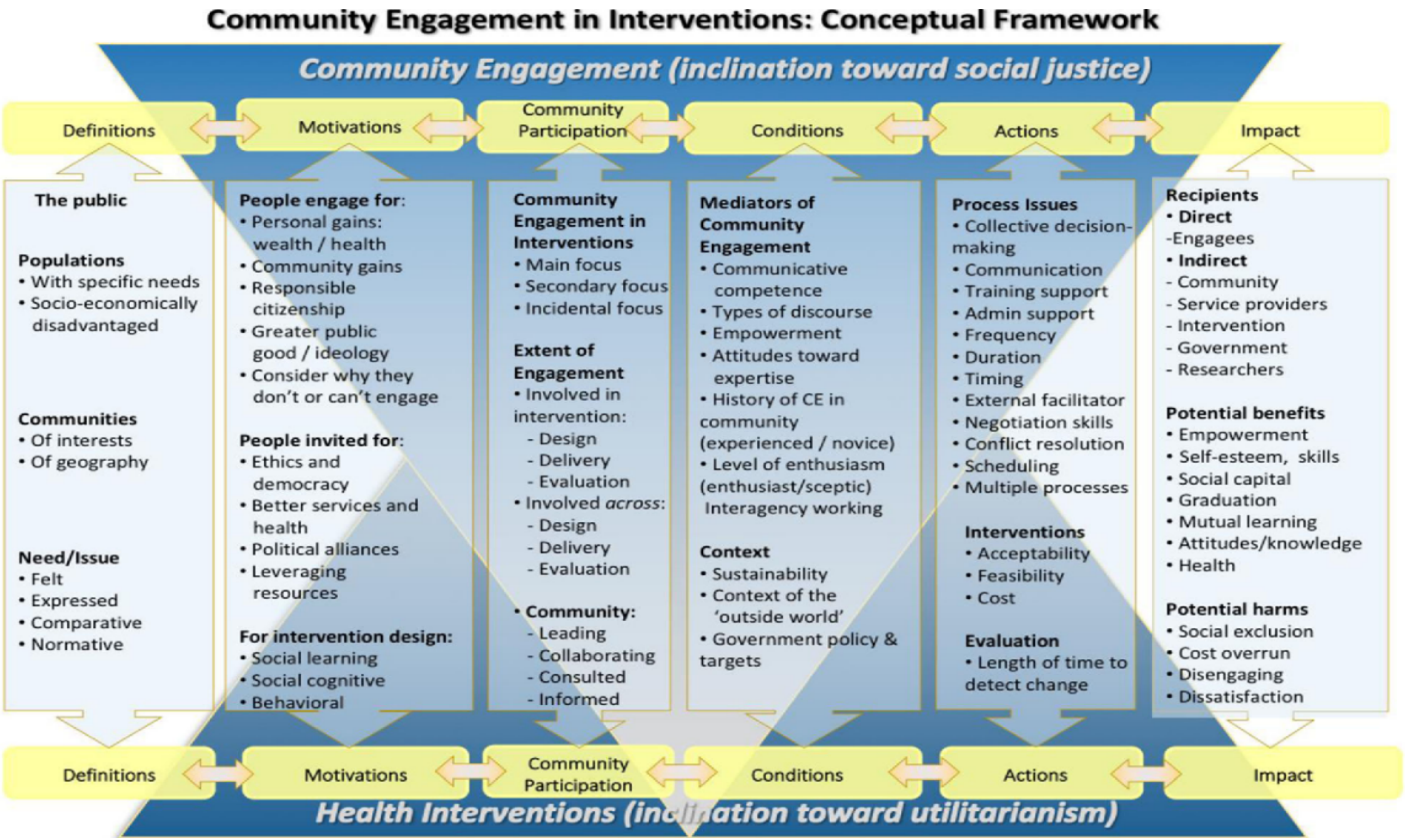

Figure 1: A conceptual framework for community engagement in interventions Brunton et al. [39]

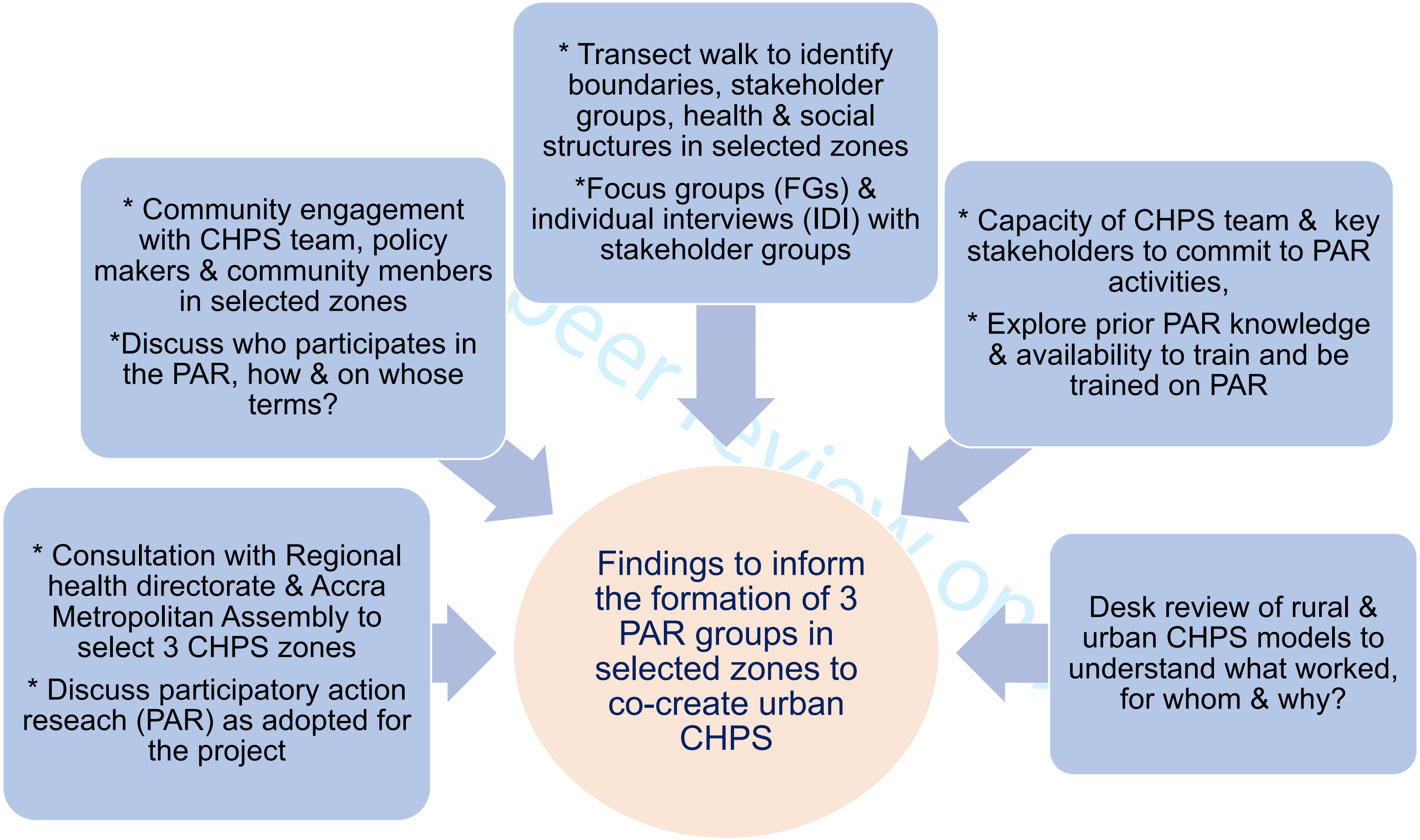

Figure 2: PAR activities in Phase 1 (Reconnaissance Phase)

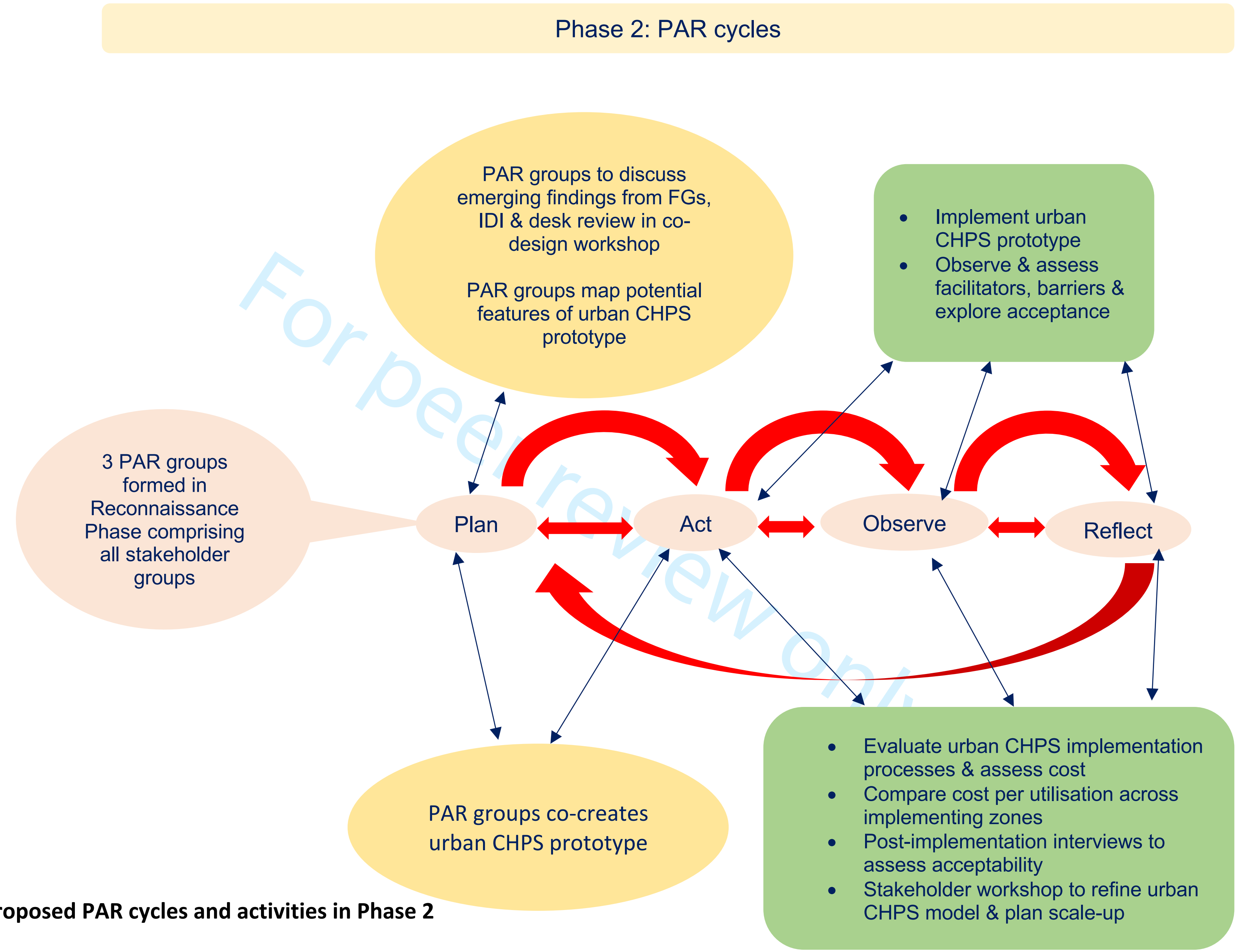

Figure 2: Proposed PAR cycles and activities in Phase 2

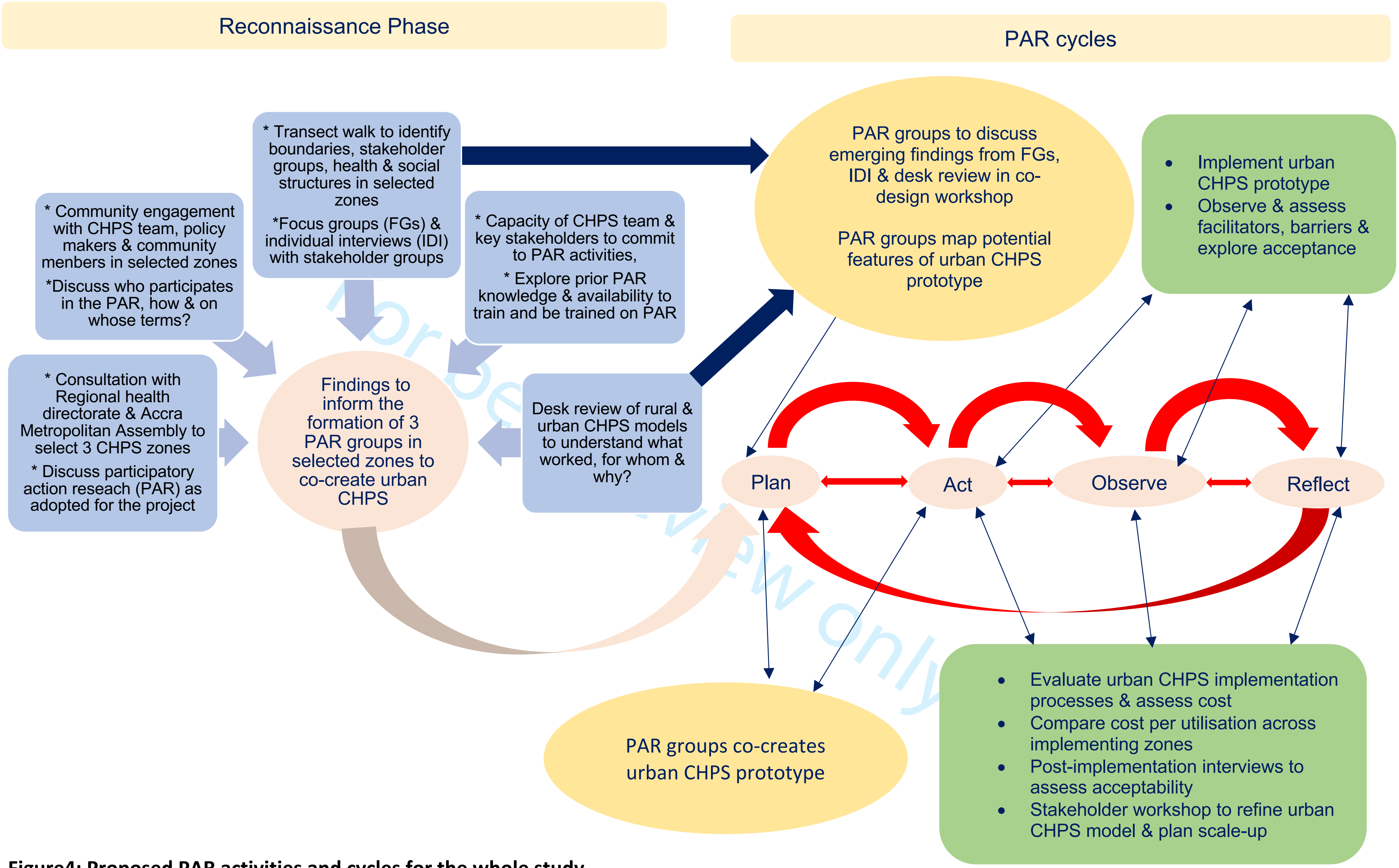

Figure4: Proposed PAR activities and cycles for the whole study
